# Micro- and Macromechanical Properties of a Composite with a Ternary PLA–PCL–TPS Matrix Reinforced with Short Fique Fibers

**DOI:** 10.3390/polym12010058

**Published:** 2020-01-01

**Authors:** José H. Mina, Alex Valadez González, Mario F. Muñoz-Vélez

**Affiliations:** 1 Grupo Materiales Compuestos, Universidad del Valle, Calle 13 No 100-00, 76001 Cali, Colombia; 2 Unidad de Materiales, Centro de Investigación Científica de Yucatán, A.C., Calle 43 #. No 130, Col. Chuburná de Hidalgo, CP 97205 Mérida, Yucatán, Mexico; avaladez@cicy.mx; 3 Grupo Materiales e Ingeniería Sísmica, Pontificia Universidad Javeriana, Calle 18 No 118-250, 76001 Cali, Colombia; mario.munoz@javerianacali.edu.co

**Keywords:** fibers, biodegradable polymers, fiber/matrix bond, ternary blends

## Abstract

Biocomposites were prepared from a ternary matrix of polylactic acid (PLA), polycaprolactone (PCL), and thermoplastic starch (TPS) and reinforced with native fique fibers from southwestern Colombia. The influence of surface modification by alkalization of fique fibers on the interfacial properties of the biocomposite was studied using pull-out tests. Additionally, the effect of short fique fibers in three proportions (10%, 20%, and 30% (*w*/*w*)) on the tensile mechanical properties of the composite was evaluated. The experimental results indicated that the interfacial shear strength (IFSS) of the ternary matrix was predominantly influenced by PCL and characterized by the development of a weak interface that failed due to matrix yielding. Furthermore, the incorporation of short fique fibers increased the elastic modulus of the composite to values similar to those estimated with the Tsai–Pagano model. The alkalization treatment of the fique fibers improved the interface with the composite matrix, and this phenomenon was evidenced by the results of the micromechanical and tensile characterizations of the composite.

## 1. Introduction

Recently, a growing interest in the research and production of biodegradable polymers derived from renewable sources, such as starches, proteins, and hydroxyalkanoates, among others, developed; these polymeric materials are environmentally friendly and are not petroleum-based (an important feature considering that petroleum is a nonrenewable natural resource), offering full biodegradability under composting conditions. Among these materials, thermoplastic starches (TPSs) stand out for their low production cost, which is based on the abundance of the basic raw material [1,2,3,4,5,6]. However, regardless of its availability and economic feasibility, TPS still exhibits disadvantages such as hydrophilicity, low mechanical properties, and retrogradation, which is a characteristic that limits use due to the recrystallization generated after prolonged storage times [7,8]. To compensate for such deficiencies, research was conducted on the use of starch-modifying plasticizers, the mixture of TPS with biodegradable polymers with higher mechanical performance, and/or the incorporation of natural fibers to obtain a biodegradable composite, such as fique fibers [9]. Despite the progress made through these efforts, continuing studies to optimize the properties of these materials and expand their applications remains important, seeing that present applications often include the partial replacement of traditional synthetic polymers in materials for flexible packaging and packing [10,11]. Based on the above, this study developed a composite material with a ternary matrix of polylactic acid–polycaprolactone–thermoplastic starch (PLA–PCL–TPS) that was reinforced with short fique fibers. Studies previous to this investigation allowed for the determination that a composition of 63%, 27%, and 10% (*w*/*w*) of PLA, PCL, and TPS, respectively, provided the ternary matrix with the best thermal–mechanical performance. This matrix was reinforced with short fique fiber percentages of 10%, 20%, and 30% (*w*/*w*) to study the effect of fiber content on the mechanical properties of the composite. Additionally, the surface of the fique fibers was modified with an alkalization treatment to evaluate the influence on the micromechanical and macromechanical properties of the composite reinforced with 30% short fique fibers. The micromechanical properties were assessed using the pull-out technique and scanning electron microscopy (SEM) micrographs of the extracted fibers. The mechanical properties were determined via tensile tests on the ternary mixture and composite materials. The results of the mechanical assessment of the composites were compared with the results obtained using different mathematical models.

## 2. Materials

The TPS was obtained using native cassava starch (*Manihot sculenta crantz*) of the variety CM 4919 with amylose and amylopectin contents of 22–26% and 78–74%, respectively. This starch was supplied by the company Ingredion from Cali, Colombia, and was processed in the form of semicrystalline particles with a spherical geometry and an average diameter of 14.400 µm. Reagent-grade glycerol, used as a plasticizer, consisted of three alcohol functionalities and was presented as a colorless liquid of medium viscosity. The extrusion-grade PLA, used as part of the matrix, was of type L (−) with a high molecular weight (120,000 g/mol), produced by Carguill Dow Polymers, LLC (Minnetonka, MN, USA) under reference 2002D. PLA had a glass transition temperature (*T*_g_) of 63 °C, a melting temperature (*T*_m_) of 145 °C, and a maximum degradation temperature of 386 °C; additionally, the polymer exhibited an elastic modulus of 3.500 GPa and a tensile strength of 53 MPa [12]. This material was purchased as translucent pellets. The PCL, used as part of the matrix, had a high molecular weight (80,000 g/mol) and was produced by Perstorp UK Limited of England (Warrington, UK), under the reference CAPA 6800. This material possessed a *T*_g_ of 45 °C, a *T*_m_ of 56 °C, and a maximum degradation temperature of 453 °C; the polymer was purchased as opaque white pellets. The fique fibers, which were used as a discontinuous reinforcement of the ternary PLA–PCL–TPS matrix, were of the genus *Furcraea* and the eagle nail (white) variety, supplied by the company Empaques del Cauca in Popayan, Colombia.

## 3. Experimental Procedure

### 3.1. Preparation of the TPS

The process to obtain the TPS was similar to that reported by Huang et al. in 2005 and by Ma et al. in 2006. Firstly, the cassava starch was dried for 24 h at 80 °C and then premixed with glycerol at a ratio of 70:30 (*w*/*w*). This process was carried out in a Black and Decker high-speed mixer for 5 min until the material showed no lumps. Subsequently, the mixture was stored in closed polypropylene containers for 72 h. Finally, the mixture was plasticized in a single-screw extruder coupled to a Brabender plasticorder model PLE-330 with a 19-mm-diameter cylinder, a 4:1 compression ratio, and L/D ratio of 25. The rotation speed was maintained at 45 rpm, and the temperature profile was 115, 125, 130, and 135 °C for the three zones of the screw and the zone of the head [1,13]. The obtained TPS was pelletized, ground, and then passed through a sieve with an average gap size of 1 mm. The test specimens were made in a Carver semiautomatic lab press and fitted with hotplates and a forced water circulation cooling system. Stainless-steel molds were used to form TPS square plates with 120 mm × 120 mm area and 1 mm thickness. For a 50-min cycle, which consisted of 30 min of heating and 20 min of cooling under a sustained pressure, the molding temperature was 160 °C, and the clamping force was 7000 lbs.

### 3.2. Preparation of the Ternary PLA/PCL/TPS Mixture

The ternary mixture was obtained using proportions of 63%, 27%, and 10% (*w*/*w*) for PLA, PCL, and TPS, respectively. These ratios were based on preliminary tests and on results reported by Liao and Wu [14]; 90% of the specified ternary mixture (which corresponded to PLA and PCL) possessed a 70/30 ratio. The mixture was prepared in the mixing chamber of a Brabender PLE-330 plasticorder station at a constant temperature of 165 °C and a spindle rotation speed of 40 rpm. TPS, PCL, and PLA were gradually added during the mixing process. A vacuum pump with a moisture trap coupled to the plasticorder was used to decrease the ambient water absorbed by the mixture. Once the ternary mixture was completed, plates were formed by compression molding using a semiautomatic Carver lab press fitted with hot plates and a forced water circulation cooling system. Finally, test pieces were machined from the molded plates to the standard dimensions required by standard ASTM D638 (“Standard Test Method for Tensile Properties of Plastics”) using a Computer Numerical Control (CNC) router.

### 3.3. Surface Modification by Alkalization of Fique Fibers

The alkalization treatment used to modify the surface properties of the fique fibers was similar to that performed by Valadez et al. (1999) on henequen fibers and that of Muñoz et al. in 2014 and 2018 on fique fibers [15,16,17]. Initially, the fibers were dehydrated at 100 °C for 24 h and, afterward, the filaments were immersed in a 2% (*w*/*v*) NaOH alkaline solution for 1 h at room temperature (25 °C). Subsequently, the fibers were repeatedly washed with distilled water partially acidified with acetic acid until neutral pH was reached. Finally, the material was dried at 100 °C for 24 h.

### 3.4. Preparation of the Composite

The composite material was made by firstly cutting the fique fibers to a length of 15 mm; this length was selected based on the preliminary processing trials, which found difficulties in producing composite materials with larger fiber sizes. The ternary mixture was reinforced with untreated short fique fibers at contents of 10%, 20%, and 30% (*w*/*w*). The preparation of the composite material was carried out in a mixing head coupled to a Brabender plasticorder at a temperature of 165 °C and 40 rpm. As with the ternary mixture preparation, the process was performed under vacuum and, subsequently, the samples were formed into plates, which were machined using a router to obtain specimens for the mechanical tests. Finally, using the same methodology followed for the composite reinforced with untreated fibers, specimens were manufactured with 30% (*w*/*w*) alkalized fibers to estimate the influence of surface modification on the composite mechanical properties.

### 3.5. Micromechanical Characterization (Pull-Out)

**Figure 1:**
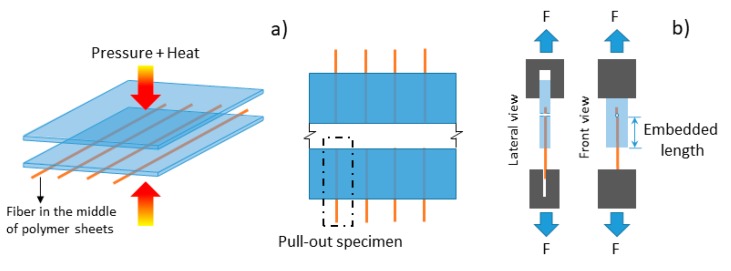
(**a**) Test specimen manufacturing, and (**b**) pull-out testing.

To determine the micromechanical properties of the various polymers tested (PLA, TPS, PCL, and the ternary mixture PLA/PCL/TPS) and the fique fibers, with and without alkalization, sheets of each studied material were manufactured. Subsequently, four fique fibers (with and without the alkalization treatment) were lined up lengthwise and sandwiched between two sheets of the same material, leaving the ends of the fibers free; thus, eight test samples were generated, able to hold and apply the necessary load for fiber extraction and/or matrix fracture (Figure 1). The sheet-fiber arrangement was hot-pressed at 150 °C and at 10% of the load applied for molding the individual plates. Finally, rectangular sections were cut in such a manner that the fiber was aligned in the middle of the specimen. The fiber extraction (pull-out) was conducted using a Minimat universal testing machine, where one end of the specimen (polymer matrix) was attached to the lower grip and the other end (fique fiber) to the upper grip. To control the length of the fibers embedded in the matrix, holes were made at different lengths (5, 10, 15, and 20 mm) in the test specimen. The test speed was 1 mm/min, and five tests were conducted for each length. Only data from specimens that did not fail due to fiber defects and/or stress induced by the grips were taken into account in the result analysis. Load–displacement curves were recorded, and the maximum recorded load was used to determine the interfacial shear strength (IFSS) as a measure of the micromechanical properties of the evaluated materials via the Kelly and Tyson equation (Equation (1)) [15].

(1) $$\tau =\frac{F_{max}}{\pi DL}$$

where τ is the IFSS, *F*_max_ is the maximum load measured before fiber debonded, *D* is the diameter of the fiber used, and *L* the length of fiber embedded in the matrix.

### 3.6. Macromechanical Characterization (Tensile)

Additions of 10%, 20%, and 30% (*w*/*w*) of fique fibers without surface treatment were evaluated to establish the effect of incorporating fique fibers on the tensile mechanical properties of the ternary PLA/PCL/TPS matrix; similarly, the ternary matrix reinforced with 30% (*w*/*w*) fique fibers with the alkalization treatment was tested to determine the influence of the treatment on the tensile mechanical properties of the ternary matrix–fique composite. To allow sufficient time for absorption equilibrium, all tests were performed two weeks after the samples were made and under temperature and relative humidity conditions of 25 °C and 54%, respectively, as done by Huang et al. (2005) [1]. The tests were conducted on a Shimadzu universal testing machine Model AG-1 100 kN equipped with a 500-N load cell. Type IV specimens and a speed of 5 mm/min were used following the standard ASTM D638 (Standard Test Method for Tensile Properties of Plastics). All measurements were taken from five test specimens, and, for purposes of analysis, the average value of the measurements was considered. Moreover, to compare the experimental results associated with the elastic modulus of the different composite materials tested and the theoretical predictions, the models specified in Equations (2)–(7) were used.

#### 3.6.1. Longitudinal Rule of Mixtures

Equation (2), allows to predict the Young´s modulus of the composite according to the properties of each of its components and the fractions of each; This applies to composites with fibers located longitudinally to the load application.

(2) $$E_{CL}=E_{f}v_{f}+E_{m}\left(1-v_{f}\right)$$

where subscripts *f* and m are associated with the fiber and matrix, respectively, $v_{f}$ is the volume fraction of the fibers, *E_CL_* is the elastic modulus of the composite calculated longitudinally to the direction of the fiber, and *E_m_* and *E_f_* are the elastic modulus of the matrix and fiber, respectively.

#### 3.6.2. Model for Composite Materials Reinforced with Short Fibers Distributed Randomly in a Plane (ECP) Tsai–Pagano 

Tsai-Pagano makes a combination of the expected longitudinal stiffness to the transversal one in ratio of 0.375 to 0.625 [18].

(3) $$E_{C}=\left(\frac{3}{8}\right)E_{CL}+\left(\frac{5}{8}\right)E_{CT}$$

where *E*_C_ is the elastic modulus of the composite, *E_CL_* is the elastic modulus of the composite calculated longitudinally to the direction of the fibers, and *E_CT_* is the elastic modulus of the composite calculated transversely to the direction of the fibers, calculated as shown in Equation (4).

(4) $$E_{CT}=\frac{E_{f}E_{m}}{E_{f}\left(1-v_{f}\right)+E_{m}v_{f}}$$

#### 3.6.3. Cox–Krenchel Model

This model modifies the rule of mixtures to fit short fiber composite materials by making corrections for the length and orientation of the reinforcements and by introducing correction factors for the length ($\eta_{l}$) and orientation ($\eta_{o}$), as shown in Equations (5)–(7) [18].

(5) $$E_{C}=\eta_{l}\eta_{o}E_{f}v_{f}+E_{m}\left(1-v_{f}\right)$$

Here,

(6) $$\eta_{o}=\underset{n}{\sum }a_{n}cos^{4}\phi_{n}$$

(7) ηl=tanh(βl2)βl2

where $a_{n}$ and $\phi_{n}$ represent the fraction of fibers with a specific orientation and the orientation angle of the *n*-th fiber; likewise, *l* is the length of the reinforcement, and *β* is a coefficient related to the stress concentration at the ends of the fibers, which is calculated with Equation (8).

(8) β=1rEmEf(1−μ)lnπ4vf

where *r* is the fiber radius, $v_{f}$ is the volume fraction of the reinforcement, and *µ* is Poisson’s ratio of the matrix.

### 3.7. Scanning Electron Microscopy (SEM)

The analysis of the surface of the fique fibers extracted from the polymer matrix during the pull-out tests was performed using SEM. Similarly, an analysis was performed on the fractured surfaces of ternary matrix composites reinforced with native and alkalized fibers, subjected to tensile tests. A JEOL scanning electron microscope Model 5400 LV operated (JEOL, Dearborn, MI, USA) at 20 keV was used. The samples were precoated with gold using a Denton vacuum coater model Desk IV to generate a conductive surface.

## 4. Results and Discussion

### 4.1. Micromechanical Properties (Pull-Out)

**Figure 2:**
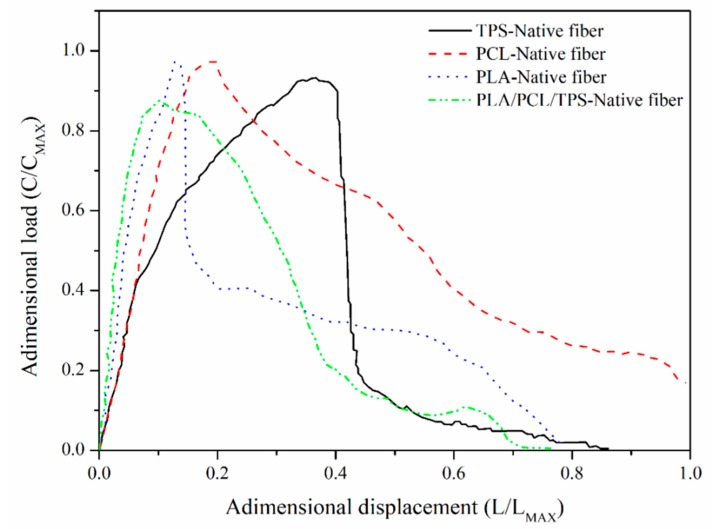
Typical loading vs displacement curves for thermoplastic starch (TPS)-native fiber, polycaprolactone (PCL)-native fiber, polylactic acid (PLA)-native fiber, and PLA/PCL/TPS-native fiber.

Figure 2 shows the characteristic loading vs. displacement curves of the ternary mixture PLA–PCL–TPS and each of its components for the pull-out tests. All materials showed a linear behavior for strains lower than 2% and a nonlinear behavior characteristic of ductile matrices for higher strains [10]. In addition, appreciable differences in the shape of the curves existed after the maximum load was reached. This result indicated the differences in the mechanical behavior of the fiber-matrix interfaces [14]. The curve for TPS with native fibers revealed that the load increased gradually until it reached its maximum value and then presented a sharp drop typical of a moderately strong interface. The PCL graph also showed the typical nonlinear behavior of a ductile matrix with a gradual drop that suggested a weak interface. Meanwhile, the PLA curve indicated the characteristic behavior of a rigid matrix with a moderately strong interface [19,20,21,22]. As for the ternary mixture, the behavior resembled more that of TPS and PCL, even though the major component was PLA; the manner in which the load dropped suggested that the interface was weak.

**Figure 3:**
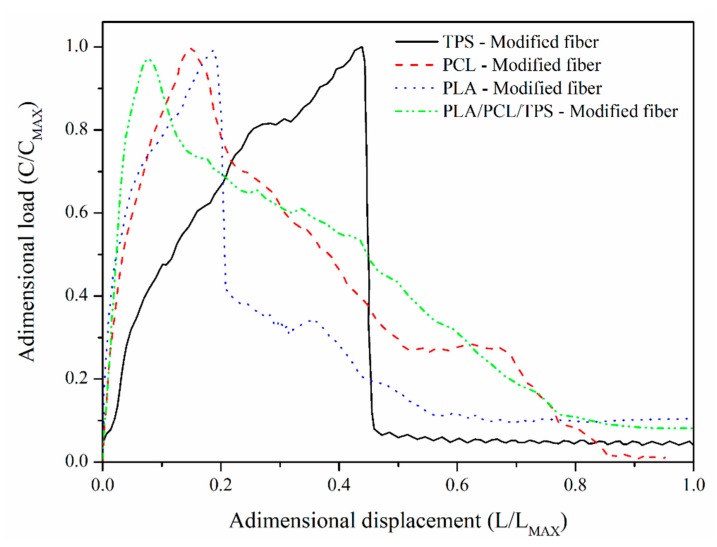
Typical loading vs. displacement curves for TPS-modified fiber, PCL-modified fiber, PLA-modified fiber, and PLA/PCL/TPS-modified fiber.

Figure 3 presents the loading vs. displacement graphs for the pull-out tests of the modified fibers (alkalized). These curves show that the main effect of the alkalization treatment on the nature of the fiber–matrix interface was a change in the failure mode. In the case of the TPS and PLA, it is evident that the interface had a brittle failure, while the interface failure mode of the ternary mixture (PCL/PLA/TPS) and PCL was matrix yielding. These results suggest that PCL plays a very important role in the mechanical properties of the ternary mixture.

**Figure 4:**
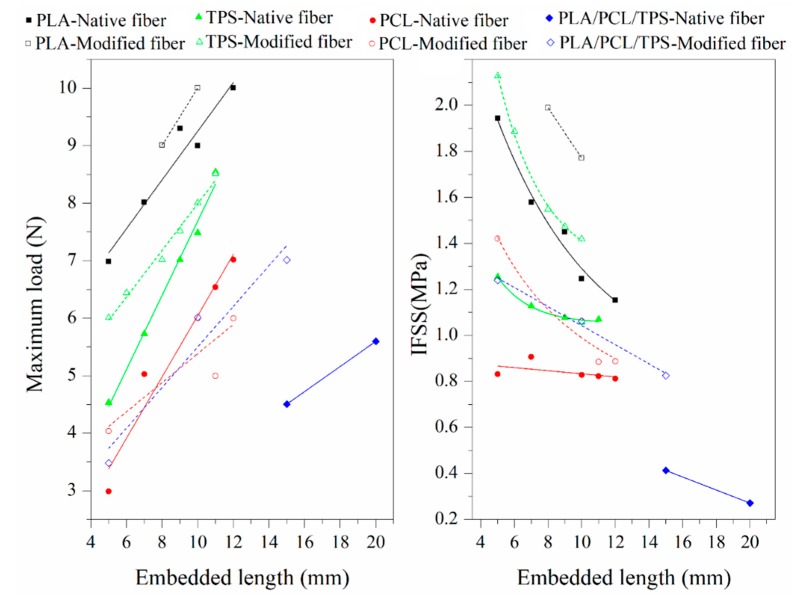
Loading curves and interfacial shear strength (IFSS) vs. embedded length for PLA, PCL, TPS, and the ternary matrix (PLA/PCL/TPS) with native and modified fibers, as evaluated by the pull-out test.

Figure 4 shows the behavior of the maximum load and the IFSS vs. the embedded length of native and modified fibers for the ternary mixture and each of its components. These graphs reveal that the load increased proportionally to the embedded length, and, in all cases, the loads were higher for specimens with alkalized fibers. Similarly, the IFSS values in materials with alkalized fibers were noticeably greater than those in materials with native fibers. Likewise, as expected, the IFSS decreased in all evaluated samples as the embedded length increased.

Furthermore, systems with PLA had the highest IFSS values, which corroborated the presence of interfaces stronger than those in the other matrix-fiber systems. It is important to highlight that IFSS values higher than those found in the PLA-fique fiber systems were expected for the TPS-fique fiber systems (with and without the surface modification). This reasoning was based on the secondary interactions of hydrogen bonds derived from the presence of hydroxyl (–OH) groups in both the TPS matrix and the fibers, which contrasts the polarity difference between PLA and the fibers, resulting in a greater incompatibility. However, the experimental IFSS results were lower for the TPS-fique fiber systems because this phenomenon could be dominated by the Young’s modulus of the matrix (3.50 GPa for PLA and 0.040 GPa for TPS) [23]; due to the above observations, in systems with TPS, the matrix was likely to deform and/or tear before slippage of the fiber. As for the PCL-fique fiber systems, the IFSS values were low, as in the systems with the ternary matrix (PLA/PCL/TPS). This result coincided with the concept stated above, suggesting that PCL has an important role in the macro- and micromechanical properties of the final composite.

**Figure 5:**
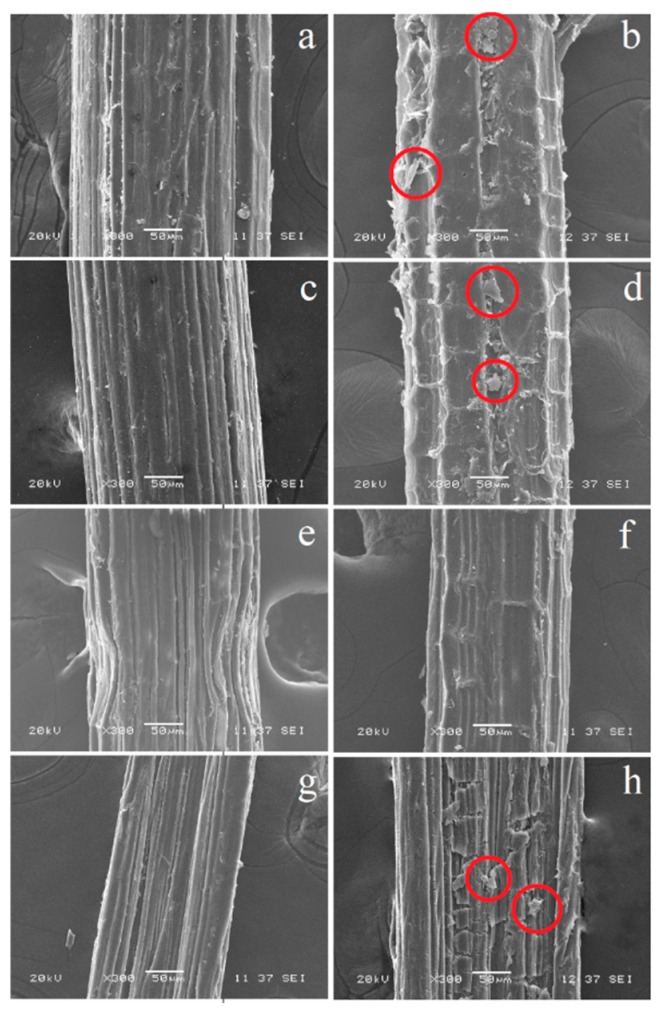
SEM micrographs of fibers extracted during the pull-out tests of the fiber–matrix systems: (**a**) TPS-native fiber, (**b**) TPS-modified fiber, (**c**) PLA-native fiber, (**d**) PLA-modified fiber, (**e**) PCL-native fiber, (**f**) PCL-modified fiber, (**g**) PLA/PCL/TPS-native fiber, and (**h**) PLA/PCL/TPS-modified fiber. The red circles show fragments of polymer attached to the fiber.

Figure 5 depicts SEM images of the surfaces of fibers extracted during the pull-out tests for TPS, PLA, PCL, and the ternary mixture. Evidently, the filaments without the alkalization treatment tended to protrude from the different matrices without showing fragments, which indicated significant adhesion at the interface, consistent with the observed behavior of the IFSS. In addition, for the specimens evaluated with modified fibers, some matrix fragments were visible on the fique fibers, which could be associated with the generation of stronger interfaces. The only exception to this behavior was seen with PCL, which essentially presented no significant matrix detachments; this result corroborated the estimated IFSS values, which were lower than those of the PLA and TPS matrices.

### 4.2. Macromechanical Properties (Tensile)

**Figure 6:**
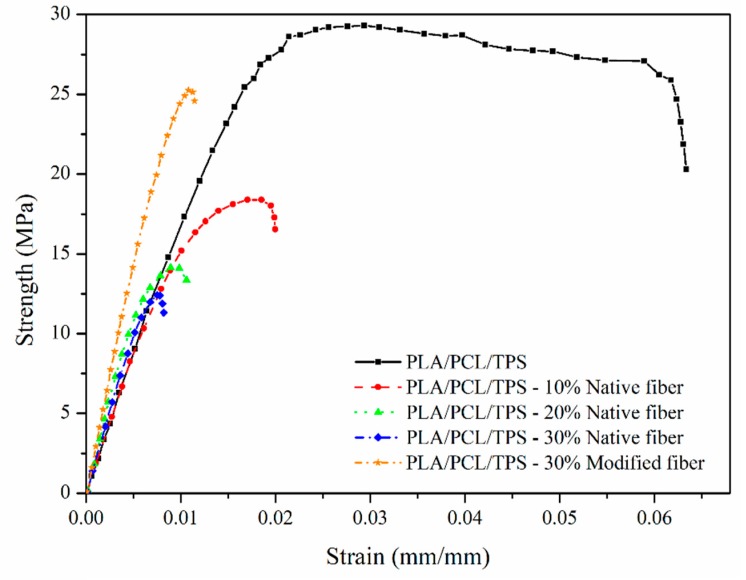
Strength vs. strain curves for the ternary matrix with 0%, 10%, 20%, and 30% native fibers and with 30% modified (alkalized) fibers.

Figure 6 shows the strength vs. strain curves obtained for the ternary mixture matrix, composites reinforced with native fibers (at 10%, 20%, and 30% (*w*/*w*)), and the composite with 30% (*w*/*w*) modified fibers (alkalinized). Here, the strength of the composite decreased with increasing fique fiber content, which was used as a discontinuous reinforcement; for all fiber fractions evaluated, the obtained tensile strengths were less than that generated by the matrix without reinforcement. This phenomenon was reported by different authors who attributed this behavior to the poor interfacial adhesion between hydrophilic fibers and hydrophobic matrices, which prevented good wetting of the fibers by the matrix; this effect increased with the fiber content in the composite [24,25]. A similar behavior was reported by Valadez et al. (1999). The authors found a decrease in the strength of a composite with short henequen fibers that were used to reinforce a polyethylene matrix; strength increases were only achieved by modifying the fibers with silane coupling agents that promoted chemical bonding at the interface [15]. These data indicate that the decrease in tensile strength with increased fique fiber content was due to a weak interface between the ternary mixture of PLA/PCL/TPS and the untreated fique fibers (native fibers).

**Figure 7:**
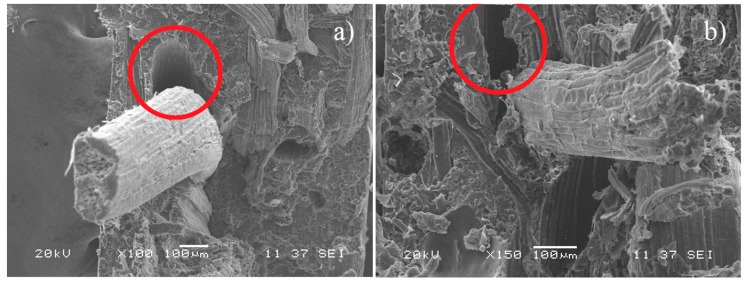
(**a**) and (**b**) show SEM micrographs of the fracture surface of the composite PLA/PCL/TPS–30% native fiber. Red circles show voids between the fiber and the matrix, suggesting a weak interface.

Figure 7 shows the micrographs of the fractured surface of the PLA/PCL/TPS matrix reinforced with 30% (*w*/*w*) native fique fibers. Apparently, the material possessed a weak interface, as characterized by fibers loosened from the matrix without showing breakage or delamination and without evidence of plastic fragments on their surface. This observation suggests that the interface did not effectively promote load transferring from the matrix to the reinforcement, concurring with the IFSS values previously estimated from the pull-out test, which, as expected, were lower than those found using alkalized fibers.

When fique fibers modified by the alkaline treatment were employed, the IFSS increased, as per the results of the micromechanical characterization. Accordingly, the composite with 30% (*w*/*w*) alkalized fibers presented a high tensile strength of 25 MPa compared with the 12 MPa found for the material reinforced with 30% (*w*/*w*) of native fibers. However, the matrix displayed yet a better tensile strength with a value of 29 MPa. In this regard, Valadez et al. (1999) also found differences of similar magnitudes between a high-density polyethylene matrix and the corresponding composite reinforced with alkalized henequen fibers [15]. Rajesh et al. (2014 and 2016) reported the tensile and flexural strengths of composites with a PLA matrix reinforced with jute fibers. The authors found that the incorporation of fibers treated by 5% alkalization did not produce a significant increase in strength; in turn, the highest increase in strength of the material reinforced with 25% jute fibers occurred with the incorporation containing 10% of fibers treated with NaOH. However, this modification corresponded to rises of only 7.7% and 8.0% in tensile and flexural strengths, respectively [26,27].

**Figure 8:**
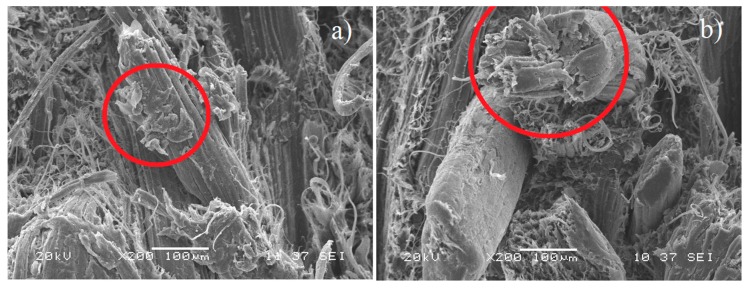
SEM micrographs of the fractured surface of the composite PLA/PCL/TPS–30% modified (alkalized) fiber; (**a**) fragments of polymers on the fiber and (**b**) fracture fiber.

Figure 8 shows SEM images including fiber fragments that evidence breaking, suggesting an efficient load transfer from the matrix to the reinforcement in comparison with the transfer to untreated fibers, as shown in Figure 7. Similarly, fragments of the matrix were visible on the fiber surface resulting from the greater mechanical anchoring caused by the roughness acquired with the surface modification. As expected, this result correlated with the IFSS values and the macrolevel properties of the material, which were improved compared to those acquired via native fibers. However, it should be noted that the properties did not improve at the expected level, and an additional surface treatment was necessary to produce a more efficient interface.

In all cases, the elastic modulus of the tested composites attained values that exceeded that of the matrix, and these increases were a direct function of the amount of short fique fibers added. The composite with surface-treated fibers gave an elastic modulus of 3.38 GPa, higher than the 2.75 GPa obtained with the material equally reinforced with 30% (*w*/*w*) native fibers. Rajesh et al. (2014) reported increases in the elastic modulus of a composite with a PLA matrix reinforced with jute fibers that were surface-modified with different percentages of NaOH. The greatest increase obtained by the authors occurred by incorporating 25% jute fibers modified with 10% of NaOH; the increase in the tensile elastic modulus was 125% with respect to the material reinforced with untreated fibers [26]. Table 1 shows the experimental elastic modulus obtained in this study, as calculated from the slope in the elastic region of the stress-strain curves of Figure 6.

With the purpose of establishing a comparison among different prediction models for the elastic modulus of the processed composites, Table 1 shows the elastic modulus calculated with the longitudinal rule of mixtures model, the Tsai–Pagano model, and the Cox–Krenchel model using Equations (1), (2), and (4), respectively. As expected, the experimental values of the elastic modulus for all percentages of fiber incorporations exceeded the modulus of the matrix (calculated at 1.768 GPa), which implies that a reinforcement effect did occur. In addition, the results revealed that the longitudinal rule of mixtures model overestimated the elastic modulus of the material. For the case of fiber-reinforced composites, the model in question was strictly applied only when the material contained long fibers in uniaxial arrays parallel to the direction of the load applied. Furthermore, the method assumed that the fibers and the matrix deformed in the same proportions, and the aspect ratio of the fiber was not considered [28].

Compared with the longitudinal rule of mixtures, the Tsai–Pagano model achieved good approximations of the elastic modulus for all processed fiber volumes. Importantly, this model took into account the aspect ratio of the fiber, which, according to Nielsen and Landel (1994), is a parameter that affects the elastic modulus of the composite material. Likewise, Hull (1987) indicated that, as the aspect ratio of the fiber decreases, the effect of the ends becomes progressively more significant because the stress and strain fields in the fiber and the surrounding matrix are modified due to the discontinuity [28,29]. To calculate the modulus following the Cox–Krenchel model, firstly, the stress concentration constant, *β*, was calculated using Equation (5) and by assigning a value of 0.300 to the Poisson’s ratio of the matrix; based on the study by Shibata et al. (2008), this datum was assumed for a similar biodegradable matrix. With this information, the length factor, *η_l_*, was obtained [30]. In addition, to facilitate the calculation, the value used for the orientation factor, *η_o_*, was 0.375; considering the experimental methodology used to prepare the composite, the fibers were placed in a random coplanar arrangement [18,29]. Table 2 shows the estimated data for parameters *β* and *η_l_*. The compatibility factor was calculated as the product of the length factor and the orientation factor, and, for 30% (*w*/*w*) fibers, the value was 0.368, which is greater than the 0.007 reported by Arbelaiz et al. (2006), for a PCL composite reinforced with 30% (*w*/*w*) flax fibers [31]. Furthermore, Ali et al. (2003) evaluated the compatibility factor to estimate the level of adhesion between pre-impregnated glass fibers and a PCL matrix, and their research found this factor to be between 0.055 and 0.191 [32]. In addition, Madsen et al. (2011) reported orientation and length factors of 0.600 and 0.500, respectively, for a composite of TPS matrix reinforced with flax fibers, which resulted in a compatibility factor of 0.300 [33]; the values referenced above are similar to those obtained in this study.

**Figure 9:**
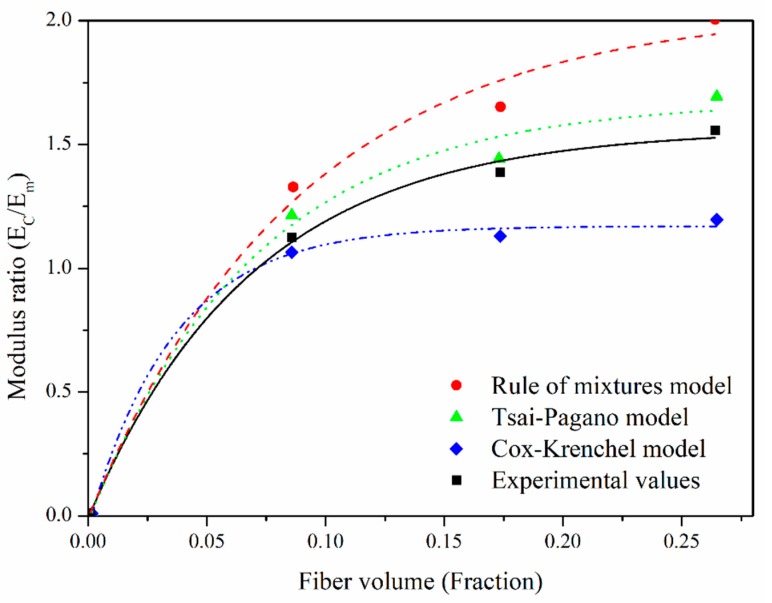
Longitudinal rule of mixtures, Tsai–Pagano, Cox–Krenchel, and experimental values.

The elastic modulus data found experimentally using the Cox–Krenchel model were lower than those obtained experimentally. El-Sabbagh et al. (2009) compared different prediction methods for composites reinforced with short fibers and found that the Cox–Krenchel estimates of the modulus were low compared with the experimental results [18]. For the data obtained experimentally and estimated by the prediction models, Figure 9 shows the curves corresponding to the ratio between the elastic modulus of the composite material (*E*_C_) and that of the matrix (*E*_m_) as a function of the volume fraction of fique fibers. It should be noted that the Tsai–Pagano model fit best to the experimental values.

The elastic modulus of the composite material with the alkali-treated fique increased from 2.750 ± 0.310 GPa to 3.380 ± 0.240 GPa for the 30% (*w*/*w*) reinforcement. This mechanical behavior was enhanced due to the increased tensile strength of the fibers after the surface modification with sodium hydroxide and to the improvement in interfacial shear strength achieved by the greater roughness of the fibers.

## 5. Conclusions

The addition of native fique fibers to the matrix formed by the ternary mixture (PLA/PCL/TPS) caused an increase in the elastic modulus of the composite; this increase was a direct function of the amount of added reinforcement. In addition, the Tsai–Pagano model fit best to the experimental data, with an acceptable degree of correlation. The quality of interface obtained in the composite reinforced with short native fique fibers was poor. This observation was reflected in the low loading values of the micromechanical evaluation using the pull-out technique and in the SEM micrographs showing regions with detached filaments but no evidence of the surface extraction of matrix fragments. With the addition of fibers, the inadequate interface eventually caused the loss of tensile strength. However, the inclusion of alkalized fique fibers improved the composite tensile strength by approximately 30% compared with that of the native fiber composite. The SEM images showed regions where the fibers exhibited fractures and a matrix present on the surface, while very few regions presented cleanly detached fibers. This observation suggested better adhesion at the modified fiber–ternary matrix interface, which was consistent with the increase in interfacial shear strength determined by the pull-out tests.

## Figures and Tables

**Table 1:** Elastic modulus of the composite material estimated with the longitudinal rule of mixtures model, the Tsai–Pagano model, and the Cox–Krenchel model, as well as the modulus calculated experimentally.

Native Fique Fiber (% *w*/*w*)	Elastic Modulus (GPa)			
	Longitudinal Rule of Mixtures Model	Tsai–Pagano Model	Cox–Krenchel Model	Experimental Values
0	-	-	-	1.768 ± 0.020
10	2.333	2.137	1.879	1.973 ± 0.208
20	2.917	2.539	1.996	2.438 ± 0.106
30	3.520	2.978	2.118	2.746 ± 0.313

**Table 2:** Parameters of the Cox–Krenchel model.

Native Fique Fiber		Fit Parameters		
Weight (Fraction)	Volume (Fraction)	*β* (mm^−1^)	$\eta_{l}$	$\mathrm{Compatibility}\;\mathrm{Factor}\;(\eta_{l}\eta_{o})$
0.100	0.085	5.210	0.974	0.365
0.200	0.174	6.310	0.979	0.367
0.300	0.265	7.440	0.982	0.368

## Data Availability

The data used to support the findings of this study are available for readers. Readers who are interested about more information could contact us at the emails shown at the top of the paper.
